# Influences on contraceptive method choice among adolescent women across urban centers in Nigeria: a qualitative study

**DOI:** 10.1186/s40834-020-00146-1

**Published:** 2021-02-16

**Authors:** Elynn Kann Sanchez, Courtney McGuire, Lisa M. Calhoun, Gwyn Hainsworth, Ilene S. Speizer

**Affiliations:** 1 Carolina Population Center, University of North Carolina-Chapel Hill, Chapel Hill, USA; 2 The Bill & Melinda Gates Foundation, Seattle, USA; 3 Gillings School of Global Public Health, Department of Maternal and Child Health, UNC, Chapel Hill, USA

**Keywords:** Contraception, Adolescents, Sub-Saharan Africa, Method choice, Qualitative

## Abstract

**Background:**

Despite calls to increase contraceptive use among adolescents and youth, large gaps still exist, creating an unmet need for family planning. Past research has focused on barriers to seeking a method. There is less understanding of the types of methods young women want and who and what influences these decisions. This study examines what method characteristics young Nigerian women prioritize when choosing a method to inform future family planning programming.

**Methods:**

In 2018, eight focus group discussions (FGD) were conducted in the Nigerian cities of Ilorin and Jos with 83 young women ages 15–24. Participants were identified by community contacts and separated into groups by religion and marital status. The discussion guide utilized a vignette structure to understand the participants’ perceptions on contraceptive behavior and attitudes and misconceptions surrounding different types of methods. The FGDs were undertaken and analyzed by collaborative teams from the University of Ibadan and the University of North Carolina-Chapel Hill. A thematic analysis of the transcripts was performed using Atlas.ti, including two rounds of coding, and multiple reviews by the research team.

**Results:**

The method characteristics associated with young women’s contraceptive decisions include: side effects, reliability, length of coverage, privacy, cost, and accessibility. Side effects, reliability, and privacy were described as negatively linked to short-acting methods whereas easy accessibility and low cost were positive characteristics of these methods. Long-acting methods were generally viewed as positive. Participants’ focus on side effects commonly resulted from concerns about the impact on future fertility. The characteristics prioritized by individuals change throughout their adolescence and as their marital status changes. Providers, peers, parents, and partners were all found to have an influence over method choice in different ways. The role of these influencers also changes over the adolescent years.

**Conclusion:**

This study demonstrates that programs should prioritize expanding method choice to increase the number of available options to ensure all young women can access a method that fits their desired method characteristics. Programming should ensure that medically accurate information is widely distributed to harness providers, peers, parents and partners as a resource for information about specific methods.

## Background

As of 2014, adolescents and youth ages 15–24 years accounted for 25% of the global population. Yet despite the size of this population, adolescents and youth experience barriers at the policy, service delivery point, and community levels affecting their access to the sexual and reproductive health services they need, particularly in low and middle income countries [1]. It is estimated that in Sub-Saharan Africa about 16% of births occur among women ages 15–19 years while 25% occur among those ages 20–24 years [2]. With maternal mortality remaining the leading cause of death among adolescent women, it is critical that adolescents and youth have access to the resources that will allow them to avoid an unintended pregnancy and/or plan for and remain safe throughout a pregnancy [1].

Nigeria, the setting for this study, faces similar reproductive health challenges as seen across Sub-Saharan Africa. Over half of Nigeria’s population is below the age of 24 years, with over half of this young population living in urban settings [3]. In 2018, the total fertility rate in Nigeria was 5.3 children per woman [3], and urban adolescents and youth have a median 5.7 year gap between first sexual experience and first contraceptive use [3, 4].

Data from recent PMA surveys demonstrate that at the national level, the prevalence of modern contraception among women ages 15–24 years was 8.8% in 2017. About a third (36%) of unmarried, sexually active young women report using a modern method while only 7% of young women in union report the same. Notably, among those young women using a method, the types of methods used vary widely by marital status [3]. Unmarried women use short-acting methods such as male condoms (56.6% of users), oral contraceptive pills (25.2%), and emergency contraception (8.4%) [3]. Among women in union, the main methods used are injectables (30.2%), oral contraceptive pills (23.0%), and male condoms (20.1%) [3]. In Ilorin and Jos, the sites of this study, the dominant methods used by adolescents and youth closely mirror these country-wide trends [5]. Despite being effective and long-acting, the use of long-acting reversible contraceptives (LARCs), such as the IUD or implant, are still limited in Nigeria, particularly among unmarried and young populations [3].

Past research has identified barriers to adolescents and youth accessing contraceptive methods. These barriers include unsupportive social norms, lack of information on methods and sources of methods, physical barriers, and youth-specific barriers like provider bias when seeking care due to their age [6–9]. However, gaps exist in the understanding of factors that influence the choice of method among young women [10, 11]. The objective of this research is to better understand how method-specific characteristics and social influencers affect method choice among adolescents and youth in two cities in Nigeria.

## Methods

This qualitative research analysis was performed using data collected as a part of a larger study on the sustainability of the Nigerian Urban Reproductive Health Initiative (NURHI). NURHI sought to increase access to and demand for contraceptive methods in urban centers of Nigeria through demand generation, service improvement, infrastructure strengthening, and advocacy. The first phase of NURHI was implemented in Ilorin, Kaduna, Ibadan, Benin City, Zaria, and Abuja from 2009 to 2015, all of which saw an increase in the modern contraceptive prevalence rate with an overall baseline percentage of 23.1% to an endline percentage of 34.2% [12]. The second phase of NURHI included implementation in Kaduna and Lagos States from 2015 to 2020 and Oyo State from 2015 to 2018. The NURHI Sustainability Study was undertaken in the cities of Ilorin (a city where programming ended in early 2015), Kaduna^1^ (a city where programming continued), and a comparison city of Jos. The study sought to understand what program elements have been sustained over time, and what barriers there are to the sustainability of programming [12]. Prior to data collection, ethical approval for the study was sought and obtained from the Nigerian National Health Research Ethics Committee (#NHREC/01/01/2007) and University of North Carolina Institutional Review Board (#17–1215). To ensure compliance with this approval, informed consent was collected verbally from each participant in her primary language, and a data management plan was followed to protect the identify of participants.

In June 2018, 18 focus group discussions (FGDs) were held with participants recruited through convenience sampling by community contacts. The focus group participants were grouped by age (15–24 and 25–39 years), religion (Christian and Muslim), and marital status (married and unmarried). To identify the social norms surrounding contraceptive use and choice, the interview guide utilized a vignette structure to understand what social norms and attitudes exist and how they either facilitate or create barriers for young women’s behavior in the community. Vignettes are a research tool that has been used in previous studies for their ability to identify community attitudes, beliefs, knowledge and social norms and create a space for the participants to share their own experiences through the characters in the story [13, 14]. For this study, the focus group participants were provided with the following vignette of a fictitious young woman at the beginning of the discussion and asked questions about perceptions of contraceptive use and contraceptive seeking behavior in their community:

*“A 16-year-old girl from your community is dating her boyfriend who is 17 years old. They decide to start having sex together. Then the girl learns that her friend is pregnant. She learns about modern contraception, such as condoms, daily pill, injectables, and implants, and is thinking about starting to use modern contraception since she doesn’t want to get pregnant like her friend.”*

After establishing and discussing this context, later in the conversation with the younger participants (15–24 years) the facilitator posed the following set of follow-up questions to gain an understanding of adolescent and youth-specific beliefs and preferences related to method characteristics and availability:

*“She seeks out advice on modern contraception from her health care provider. Her provider recommends two options: combined oral contraceptive pill, which she must take each day and can purchase from a patent medicine vendor (PMV) or a Pharmacy, or an implant, which is inserted [by a provider] in a health facility and is effective for 3-5 years and can be removed at any time if she wants to become pregnant.*

- *Which method do you think she will choose?*
- *Why would she choose that method?”*

The participants’ perspectives and knowledge on contraceptive method choices throughout the FGDs are captured in the results below. The majority of the results were in response to this extension of the original vignette. This analysis focused on the FGDs with participants ages 15–24 years. In total, this analysis analyzed eight FGDs in the two cities with nine to thirteen participants each, equaling 83 participants whose demographics are included in Table 1.

**Table 1:** Focus group discussion demographics: participants ages 15–24 years

FGD number	Location	Marital status	Religion	Number of participants	Average age of the participants (by city)
1	Ilorin	Married	Christian	12	20.4 years
2	Ilorin	Married	Christian	9	
3	Ilorin	Unmarried	Muslim	9	
4	Ilorin	Unmarried	Christian	13	
5	Jos	Unmarried	Christian	10	20.9 years
6	Jos	Unmarried	Muslim	10	
7	Jos	Married	Muslim	10	
8	Jos	Married	Christian	10	
Total				83 (10.4 average)	

The FGDs were led, transcribed, and translated by members of the University of Ibadan Centre for Population and Reproductive Health (CPRH). The resulting transcripts were shared with the research team from the University of North Carolina-Chapel Hill’s Carolina Population Center. A thematic analysis was performed that began with reading the transcripts while creating notations of the emerging themes. These themes were combined with research question specific codes to create the original codebook that was uploaded to Atlas.ti and applied to the transcripts in the first round of coding. A second round of coding was performed to incorporate emerging themes that were not captured in the first codebook. Atlas.ti’s analysis tools and an Excel matrix were utilized to identify trends across subgroups using the themes captured through both rounds of coding. A specific analysis was also performed to look at mentions of specific contraceptive methods in comparison to comments about method features generally.

## Results

This analysis identified important influencing features that adolescent women consider when they are selecting a contraceptive method. The presence or absence of side effects and concerns about the impact on future fertility were the most frequently discussed factors influencing method choice, with the reliability of the method closely following. Lifestyle fits such as privacy, cost, and accessibility were also considered as influential in the overall decision of what method to choose. These influences were attached throughout to specific methods with short-acting methods being more likely to be associated with features that would detract from use, while implants were more frequently associated with features that would encourage use. Participants discussed providers, peers, parents and partners as people who would play a role in influencing the character’s decision, though they described these roles differently.

From the start of each discussion, it was clear that consideration of contraceptive choice existed within a larger context where sexual activity and contraceptive use broadly was not fully supported for adolescent populations. The resistance to the character’s sexual activity was inherently associated with the character’s use of a contraceptive method, creating hesitancy toward talking about certain methods. This was particularly true when discussing unmarried adolescents, as a young woman’s marital status served as an important filter by which to view her actions around sexual activity and contraceptive method use through. However, once the participants acknowledged likely contraceptive use due to the storyline of the vignette, the participants discussed how the predominant influences are considered within this social context. Throughout this results section, the influencing factors will be discussed starting with the most frequently mentioned (side effects) through the least frequently mentioned (accessibility). Then we will more thoroughly describe the influencers of a young woman’s decision on what method to use. When appropriate, the regional or demographic differences in responses will be included.

### Method specific features

#### Side effects

One of the most widely discussed reasons for contraceptive choice was the potential side effects caused by a method. The broad term, ‘side effects’ was the most frequently mentioned term by participants, while, less frequently, participants discussed specific side effects including loss of menstruation and weight gain.

A method’s impact on the character’s future fertility was widely discussed across all but one of the FGDs. However, any quantification of these discussions would result in a conservative estimate as the young women’s general use of the word “side effect” may have also been referring to fear of infertility. For example, participants never specifically mentioned a delay in the return to menstruation after stopping a method as a concern, though in previous studies, it has been identified as a concern by women of all ages, specifically with injectable [10]. While this side effect was not stated as a concern specifically, it would fall within the scope of “concerns around future fertility.” Lack of probing on the distinction between general and specific side effects may have led to this potential discrepancy.

The participants’ prioritization of avoiding side effects in the long term was illustrated by an unmarried, Muslim, young woman from Ilorin who pointed to side effects as the reason for her resistance to contraceptives broadly in this interaction with the moderator,

*“Participant: I think it is not good for her because she is too young.*

*Moderator: In this situation, are you saying it is not good for her to use modern contraceptives?*

*Participant: Yes, because it could have side effects when she’s grown up.”*

There were participants in all of the FGDs that expressed the general lack of support for contraceptives exhibited by the participant above. However, in this example, it was only when probed that side effects were described as the reason for this general concern. Additionally, this participant’s use of the word “side effects” in the long term supports the possibility that participants’ use of “side effects” could truly be connected to fertility concerns.

For some young women, concerns about side effects were not attached to a particular method, like one married, Christian, young woman from Jos who said,

*“I will not advise her to go for it [contraceptive use] because, in as much as they are safe to be used as contraceptive that is used for preventing pregnancy, they all have their side effect … ”*

This young woman makes her recommendation based on prioritizing avoiding side effects over pregnancy prevention, a sentiment shared by many. The most frequent reason condoms were recommended as a contraceptive method was due to their lack of side effects. Participants commented on the side effects of specific methods, including much discussion of oral contraceptive pills; the discussion revolved around infertility and other side effects of oral contraceptive pills, including irregular bleeding and weight gain, in over half of the FGDs. Side effects of implants were also infrequently mentioned.

#### Reliability & length of coverage

Method reliability was a method-specific feature mentioned frequently by participants and framed as the general likelihood for user error, not the specific failure rate once used. For example, participants commonly described that oral contraceptive pills can be easily forgotten, or highlighted barriers to getting another injection, which in turn creates an opportunity for unintended pregnancy. Participants referred to the process of taking oral contraceptive pills daily as “unreasonable,” like this unmarried, Christian, young woman from Ilorin, who responded to the moderator’s question about if the character would choose the implant or oral contraceptive pill by replying,

*“I think me as a person, I will advise her to go for the other one in which they will inject because you taking pills every day it's not reasonable and then sometimes something might happen probably forget or something might happen.”*

Like this example, participants were more likely to indicate that they believed the character in the vignette would choose the implant because once placed, it could be forgotten until she wanted to become pregnant. In five of the eight discussions, participants directly talked about the effectiveness of the implant, and their trust in the method was discussed in combination with the implant’s simple insertion. An example of this was when an unmarried, Christian, young woman from Jos shared her support for the implant by stating,

*“To me, it’s better she uses the implant because she can remove it at any time that she feels like and as far as it is in her body, she won’t get pregnant but she can remove it when she wants to get pregnant.”*

The number of participants that recommended the implant to the character indicates that participants prioritized selecting a method with a low potential for user error, which they also connected with a low level of interaction with the method itself.

Connected to this, we see that this participant and others valued the length of time that the implant would provide pregnancy protection, while also linking that length of coverage with flexibility. Participants commonly framed their discussions on the length of coverage around how often it would require them to interact with their method or seek care. As discussed above, participants commonly found taking oral contraceptive pills daily to be a concern, while acknowledging that it might be hard to access a new pack once the first pack was finished. Alternatively, the length of coverage provided by the implant’s 3–5 year lifespan limits the participant’s interaction to just the insertion and removal, which many viewed as a positive feature of the method. In two of the FGDs, participants showed support for the 3-month length of coverage provided by an injectable, though a few participants did discuss the challenge of remembering when to go for the next shot. Participants discussed flexibility with a method both through the frame of not having to interact with the method as well as being able to quickly decide to stop using the method should reproductive priorities change.

#### Privacy

The prioritization of privacy, meaning that others do not know about her contraceptive use, was a factor that was considered by the participants for all discussed methods, especially when there is a lack of social support for a young woman seeking a method. All of the participants who discussed privacy did so with the concern that the character’s parents would find out about her method use. These mentions associated oral contraceptive pills negatively with privacy, while implants were positively connected with this factor. For example, one unmarried, Christian participant from Ilorin said,

*“If I’m to recommend for her, I will surely want her to go for the implanol [implant] because you said it will be inserted around her arm and obviously it will be small it won't be something heavy, she could always put on a long sleeve unlike her taking pills every day. It's absurd and at a point, the parents will want to know what the drugs are for.”*

The participant indicates that the implant allows for the character to use a method covertly while avoiding the social pressures that accompany other methods. There were no observed differences in mentions of privacy across marital status or religion; however, mentions were concentrated among participants in Ilorin.

#### Cost

Unlike other factors, there was a divide in the participants on if the cost would be a consideration for the character. The cost of a method was raised in half of the discussions as a potential barrier, while the cheap price of oral contraceptive pills was mentioned as a benefit of the method less frequently. A few participants did acknowledge that the one-time payment for a long-acting method may be preferred, like one married, Christian participant from Jos who said,

*“that one [implant] will be the easiest because she will just put it there and it will remain there as much as she wants and there will be no problem, but that other one will be stressful for her going to buy it, if there is no money it will be another problem.”*

For this participant, shorter-acting methods require a young woman to have consistent access to money. For adolescents and youth, the cost of oral contraceptive pills can be what initially attracts them to the method; however, this participant is arguing that the monthly payment may also be a disadvantage.

Alternatively, moderators in almost all of the FGDs specifically asked if the cost would be a barrier to accessing a method, and participants widely indicated that it was not. As one married, Christian, young woman from Jos said, *“as far as she is desperate, to have it, she would at any cost, she would go for it.”* This participant is making a general statement that cost will not be a barrier due to the girl’s determination. Additionally, other participants indicated that a partner would pay the cost. There were no differences in these beliefs between group demographics (marital status and religion), indicating that the role that cost plays in a young woman’s decision varies widely depending on the individual young woman’s situation.

#### Accessibility

The final factor that a handful of participants mentioned as a benefit of short-acting methods was that they were easily accessible. Participants discussed the accessibility of condoms and oral contraceptive pills, both of which can be purchased at the local pharmacy. For example, in this exchange with the moderator, an unmarried, Christian participant from Ilorin raises two main reasons for her belief that the character will choose the oral contraceptive pills,

*“Participant: the implanol, it will have to be implanted in her so she will feel it will be more difficult than the pill she will feel it's easier and of non-stress. So she will want to go for the pills*

*Moderator: So you are saying that she will go for the pills because it's easy?*

*Participant: yeah it is easier disputing the fact that she's going to take it every day but the implantation stuff, she will feel it's too dangerous or something that's somehow for her to do it because you have to implant it then if you want to remove it, you still have to remove it.”*

In this interaction we see the participant talks about accessibility in two different ways. First, she supports oral contraceptive pills for the character’s ability to conveniently access them within her community. But we also see another idea of accessibility, which is that this quote appears to reflect fear surrounding the insertion process and provider bias which could also be a factor among this population [6]. Other participants supported this sentiment and indicated that in an environment where contraceptive use for young people is not fully supported by societal norms, being able to access oral contraceptive pills and condoms outside of facilities could be a supportive factor for these short-acting methods. It is important to note that this participant’s mention of fear surrounding the insertion process of an implant was one of only two mentions of this concern. If this fear is a barrier for adolescents, it was not widely discussed in these FGDs.

### Influencers of method selection

#### Provider

The most frequently mentioned influencer on the character’s method choice was a doctor or nurse, as participants mentioned them more than twice as often as any other influencer throughout the FGDs. These mentions were intrinsically linked to the value participants placed on avoiding side effects. Participants saw medical professionals as a source of information on methods that would best “fit one’s blood,” with some young women discussing tests that could be run to find the best option. Others discussed how doctors could give information on multiple options from which the character could choose. An unmarried, Christian, young woman from Ilorin shared one example of this idea,

*“ … she can seek medical advice because when she gets there she will know the different types of pills they use that can help her, that will not damage her womb … ”*

This participant reflects the overwhelming sentiment that the avoidance of side effects is a key influence on a young woman’s decision when selecting a method; a process a provider can help to inform.

That said, the influence that doctors and nurses have on a young woman’s decision was framed quite differently than the role of other influencers such as friends or family members. Unlike other influencers, the character’s decision to seek care at a hospital or clinic was discussed as something she *should* do versus something that she *would* do. For example, one unmarried, Muslim participant from Jos said,

*“I think she should go and see a doctor, they are the ones that will lead her on family planning so they can check which is better for her.”*

Within the context of the participants’ prioritization of avoiding side effects, this language on what the character should do is in line with the community’s priorities. However, it does raise the question of if the participants’ responses reflect what they think the character would actually do. On average, unmarried participants were three times less likely to discuss the role of a provider on influencing the contraception decision than married participants; this hints that there may be barriers to visiting providers that went undiscussed in the FGDs. This is supported by 2017 PMA2020 data that found that in Nigeria only 6.7% of unmarried adolescent family planning users accessed a method from a public facility as compared to 28.4% of married adolescent users [4]. It is notable that while pharmacies and patent medicine stores can be a place to access contraceptives without having to see a provider, only a small number of participants mentioned these as an option for young people. The majority of the FGD moderators did ask whether the character would face any challenges during a clinic visit. A few participants indicated that she would face some provider bias or disqualifying questions because of her age. However, this sentiment was not expressed widely, with many participants disagreeing that these types of challenges would occur.

#### Peers & parents

Peers were referenced almost two times more than parents, though both were discussed as playing a similar role in providing information to the character. While providers were discussed as helping the character find the method that would be the best medical fit through tests, peers’ influence on method choice came in the form of stories that relayed information about characteristics of different methods as experienced by themselves or friends. It was quite common for participants to anticipate the character’s friends to use personal experiences as a way of introducing these preferences of a method. For example, one unmarried, Christian, young woman from Jos stated,

*“Yes her friends can influence her because maybe they will say I’ve used it, oh and it last so you can also go for it so it will also last.”*

This short example shows how the speaker grounded her response in her perspective of friends sharing stories. In another example from Jos, a married, young woman describes general side effects from using contraceptives.

*“It really has so many effects, many people bleed a lot. Those who have received it are those who tell us that they did it and it was not a pleasant experience, so they give advice. That is why people refuse to do it because there are so many side effects.”*

This example echoes many others, like a young woman from Ilorin who had a friend who gained weight on oral contraceptive pills, or a young woman from Jos whose sister developed low blood pressure after receiving an implant. As the participants shared the stories that they had heard from friends, it indicated that these stories directly influence one’s views on certain methods. Though this can be positive, the majority of these examples throughout the FGDs were about negative experiences with methods. The misconceptions about side effects as well as real side effects experienced by individuals are shared within peer groups, which can feed into the fertility concerns discussed above. Overall, participants saw peers’ experiences as an introduction to new methods and as a litmus test for their safety.

Parents were discussed in a similar role, as people the character could turn to for stories (both positive and negative) about certain methods, in turn influencing views of the mentioned contraceptive. Additionally, parents were described as being able to bridge the gap between the character and a provider by bringing the character to a clinic. However, some participants cited privacy concerns in discussions about parents due to some parents’ resistance to contraceptive use. In this way parents were seen as potentially wanting to influence the character away from use, leading to a young woman not seeking a parent’s advice if she wanted to avoid this pressure. Similar to the described influence of providers, seeking support from parents was more likely described as something the character should do, versus would do. Because of this, participants indicated that the influence of parents is complicated and dependent on the type of relationship that each young woman has with her parents.

#### Partners

Partners were not discussed frequently as directly influencing contraceptive decision-making, however, when mentioned, their reference highlighted a different type of influence on a young woman’s method choice than that of the provider, peers or parents. Partners were discussed as potentially covering the cost of a method. Additionally, a few participants mentioned that partners might have a preference over the type of method that is chosen. For example, in every FGD, participants discussed that a male partner would not want to wear a condom, particularly if a young woman was using another contraceptive method for pregnancy prevention. One group highlighted the risk of sexually transmitted infections associated with this trade off, however, this concern was not discussed widely by the groups.

Though this may not always be the case, when partners were mentioned, they were discussed as influencing the character’s method choice decision by playing a key role in making the decision for her, while other influencers were simply providing information that would lead the character to a certain decision.

### Navigating pathways

How one prioritizes different qualities of a method may vary depending on one’s current physical and social situation. Further, the method chosen may change over time. This idea of method fluidity is particularly well captured by an unmarried, Christian, young woman from Ilorin who, in the below exchange, highlights a young woman’s changing method choice over time:

*“Participant: I think for starter, she will get a cheap one [family planning method] then maybe later she might upgrade and go for the expensive ones that will last longer.*

*Moderator: some long-lasting ones are actually free. So what do you think she will go for? Is it because they are easy, I am not talking about money, I'm talking about easy to get and easy to use or injectable, you will go and get injectable after two months or three years or five years. Which one do you think she will use?*

*Participant: that's what I’m saying, for starter she might get the easier ones [pill or injectable] then maybe later when she's free and have enough time or when she's confident enough to go to the hospital then she will go for the ones that will stay longer.”*

The wide range of factors that participants discussed through the FGDs closely mirrors this quote, as the participant acknowledges that each person will have a different feature that they are prioritizing when choosing a method. These priorities will also change for an individual with experience, resulting in different method choices as individuals ages or goes through life changes. Throughout these discussions, the participants discussed cost, reliability, length of coverage, and accessibility. While side effects and future fertility are overwhelmingly important factors for adolescents and youth, young people will also factor in accessibility and cost when making a final decision.

## Discussion

Overall, the results of this study find that short-acting methods, specifically oral contraceptive pills, were more frequently associated with factors that would deter from use, though were discussed as the more accessible and affordable method option. Alternatively, long-acting methods were discussed more positively and were preferred among respondents who desired a method that required little follow-up at health facilities and was less user-dependent. Providers, peers, parents, and partners were all found to have an influence over the character’s method choice in different ways, highlighting that successful interventions for young people require the support and mobilization at multiple levels.

The results indicate that young people prioritize a sense of control, which can look different to different people. For example, oral contraceptive pills were frequently associated with convenience and cost, both of which a young person may see as factors they can control, while the majority of participants indicated that a method that was reliable and long-lasting was preferred, giving them greater control over their reproduction due to the effectiveness. The role of the provider also highlights this sense of control as participants wanted the character to gain trustworthy information about methods to make an informed decision. Both parents and partners were discussed with hesitance as both parties were mentioned as potentially taking the control out of the character’s hands, with unsupportive parents limiting access while partners were described as the decision maker in the choice of method. Having control of one’s own reproduction was a priority for the participants and ensuring access to an expanded choice of methods ensures she has the control to choose the method that would be the best fit.

This study did find some misunderstandings around the proper usage of the oral contraceptive pill. There were multiple occurrences of participants believing that oral contraceptive pills only need to be taken when the young woman wants to have sex, and this inaccurate feature was highlighted as a positive characteristic related to the method’s flexibility. This potentially could be due to a confusion between the oral contraceptive pill and emergency contraception, a distinction that was not made in any of the discussions. Some participants did acknowledge that for young women who are accessing their short-acting methods through drug shops, they may not be receiving all the proper information about the method, or potential side effects. The improper use of short-acting methods can lead to side effects and unplanned pregnancy, which are outcomes that may be discussed with friends and dissuade them from using these methods in the future. This possibility for misinformation must be addressed with programming that ensures the distribution of medically accurate information. The WHO has resources available that relay the most current information on each contraceptive method, and how to properly counsel someone when choosing a method [15, 16]. Many of these resources focus on providers, though they should guide the creation of community materials and programming to ensure this information is distributed more widely.

Our results are supported by global data which finds that unmarried young women are more likely to use traditional methods or condoms while married young women are more likely to access methods such as injectables; LARC use is still low among both groups [17]. Nigerian PMA2020 data from 2017, supports these usage trends as well, and the factors identified by the participants in this study highlight why certain individuals are drawn to certain methods [3]. One area where the results diverged from global research was the dominance of providers as reported influencers when looking at the data collectively. Despite the support by the participants, previous research shows providers can play a restricting role on access to the use of contraception [5]. Our results did potentially support these restrictive roles when the data was analyzed by marital status, though direct mentions of provider restrictions within the data were limited. The findings around peers align with previous work. For example, one study among Nigerian youth found that 85% of female participants interviewed based their contraceptive decisions off of their peers [17]. The findings of our study were also in line with previous research about method-specific features that are desired by young women. Two previous studies found that participants ranked a lack of side effects and effectiveness as the most important features for them, which was supported by our findings though a lack of side effects and concerns of future fertility were the most mentioned factors among our participants [10, 11]. That said, privacy, which has been identified by several other studies as a concern, was minimally discussed by our participants [11, 18].

This study has some limitations as a result of the use of a vignette structure in the discussion guides. Vignettes are a form of priming and can have two possible influences. First, the vignette sets the scene that the character is accessing a method at a doctor. Due to this framing, we had limited ability to analyze participants’ views on pharmacies and drug shops as they were not frequently discussed in the FGDs. Though young women can face barriers of negative social norms around adolescent and youth use of contraception and provider bias at a facility as well as a pharmacy or drug shop, proximity, and other convenience factors may lead young women to access contraception at these alternative non-health facility-based locations if choosing between a health facility and a pharmacy or drug shop. However, the findings of this study are unable to determine if this is the case. Because of this limitation, a more open-ended analysis of the pathways to a method should be considered for future research. Additionally, in the follow-up questions, by limiting the choices for participants to choose from to oral contraceptive pills or an implant, it limited the responses that participants provided on their believed method the character would select. Based on the Nigerian PMA2020 data on adolescent method mix, we know that implants account for only 14.4% of use among married adolescents compared to 30% for injectables [6]. Among sexually active, unmarried adolescents, these numbers fall below 5% for both methods, with condoms accounting for over 50% of use [3]. Additionally, the 2013 Nigerian Demographic and Health Survey found that among all women surveyed, only 24.7% had knowledge of implants while 70.9% had knowledge of pills [4]. By limiting the methods the participants were meant to discuss, it created an image of high implant use that is not supported by population data. This approach provided more detailed look at these two methods that offered perspectives on hormonal methods that required daily reminders versus a longer-term, less user dependent method. This study demonstrated an interest in certain features of implants or LARC methods, but this may ignore the fact that for young people these methods are potentially inaccessible. Future research would be needed to determine whether the use of LARCs among young women would increase if availability and accessibility of these methods was ensured.

## Conclusion

The results of this study can be used by programs seeking to increase expanded method choice for adolescents and youth. In particular, this study demonstrates that young people are concerned about side effects and health effects and programs need to address these issues as part of strategies to increase use in this population. Further, programs should continue to prioritize expanding method choice by ensuring that accurate information is available on all available methods and addressing misinformation about methods that may cause hesitancy toward use, or use of some methods in certain contexts. Providers, peers, parents, and partners can be harnessed as a resource for information about specific methods and this may lead to these influencers being more supportive of young people’s contraceptive use. Programming should ensure that medically accurate information is widely distributed to potentially reach these influencers and that providers are trained on youth friendly services that includes provision of a range of methods to young people. Further, promoting parent-child communication as well as normalizing contraceptive use among sexually active young people can help reduce barriers to use for this group.

## Data Availability

The datasets generated and/or analyzed during the current study are not publicly available in order to protect the identities of the participants involved but are available from the second author on reasonable request.

## Footnotes

1 FGD were also held with young women in Kaduna, however, relevant questions required for this analysis were not included in the Kaduna FGD. For this reason, Kaduna data are not included in this analysis.
